# An exploration of the feasibility of radiation therapist participation in treatment reviews

**DOI:** 10.1002/jmrs.23

**Published:** 2013-09-02

**Authors:** Clare Maree Monk, Stephanie Jane Wrightson, Tony Neil Smith

**Affiliations:** 1 Department of Radiation Oncology, Calvary Mater NewcastleNewcastle, New South Wales, Australia; 2 Department of Rural Health, University of NewcastleTaree, New South Wales, Australia

**Keywords:** Advanced practice, medical intervention, radiotherapy, review clinics, workforce reform

## Abstract

**Introduction:**

As radiation oncologists' (ROs') workload has increased over time, treatment review clinics have become recognized as an area of RO practice into which radiation therapist (RT) practice could extend. There has been limited utilization of RTs in this role in Australia and a paucity of data on the acceptability and opinions regarding RTs practising in this role in an Australian context. The purpose of this audit was to investigate the feasibility of RT participation in review clinics at Calvary Mater Newcastle.

**Methods:**

Feasibility was determined by two methods: an audit of 200 treatment reviews to determine medical intervention (MI) levels required and a survey of 80 clinical staff to explore attitudes towards RT participation in clinics.

**Results:**

Medical intervention was required in 59% (*n* = 118) of observed reviews, with the lowest being for breast (33%) and prostate (28%) cancers. MI peaked at 73% between fractions 16–20 and was lowest early and late in the treatment period at 48%. There were 60 responses to the staff survey. All but one respondent agreed that RTs would be willing to participate in treatment review clinics, but all five consultant ROs indicated they would not be willing to delegate reviews to RTs.

**Conclusions:**

Neither feasibility measure reached acceptable levels to recommend RT participation in treatment review clinics. Further investigation and RT education are required to help meet the future RO workforce shortfall. As MI rates are lowest for breast and prostate cancer RT participation could be targeted to these clinics.

## Introduction

Patients undergoing radiation therapy attend treatment review clinics where reviews are performed by radiation oncologists (ROs). Treatment reviews provide the ROs an opportunity to monitor and manage potential side effects and to provide clinical and psychosocial support to patients. Additionally, there is a quality assurance component to ensure that treatment is progressing as planned. Depending on the treatment site and fractionation, patients can have as many as eight scheduled review clinic appointments with their RO during the course of their treatment, which contributes substantially to ROs' workload. As ROs' workload has increased over time, largely due to the ageing population and increasing number of patients requiring treatment, it has been recognized that review clinics could be delegated by ROs to other health professionals, including radiation therapists (RTs). This has potential to promote a more integrated, team-based approach to patient care.^1^

RO workload has increased substantially over the last two decades, as has the complexity of technology and treatment protocols. Between 2000 and 2010, the number of ROs in the Australian workforce increased by about 76%^2^,^3^; however, according to Medicare statistics, the number of radiation oncology services increased by 141% over the same period.^4^ The annual number of services is projected to increase rapidly with population ageing, while it is unlikely that the growth in the RO workforce will occur even at the precedent rate. Growing recognition of this mismatch has led to the evolution of new practice models that involve the delegation of roles and responsibilities from ROs to RTs.^5^

With much initial work done in the United Kingdom,^5^,^6^ RT participation in review clinics has been in practice since at least 2000 in some parts of the world in the context of the development of advanced practice models. Reported benefits include increased communication, decreased patient waiting times, and more consistent patient monitoring and management.^6^–^8^ RO time and workload is saved and can be utilized to assess more complex patients or to perform other higher level duties. The increased role for RTs has potential advantages of improved job satisfaction and better recruitment and retention.^1^ Data have shown that some patients present to clinics with minimal treatment-related sequelae, resulting in a low medical intervention (MI) rate occurring in clinics.^7^–^11^ This low need for MI further enables RT participation.^7^–^11^

Patients have indicated they are receptive towards RT reviews.^7^–^12^ In one study of 865 breast cancer patients, 97.6% were happy to not see their RO during treatment and 99.7% were highly satisfied with time spent with the RT review staff.^10^ Another study of nurse-led treatment reviews of head and neck cancer patients showed they had no preference towards their RO leading review clinics.^13^ With proper clinical training and advanced education, both RTs and ROs have concluded that reviews in clinics can be effectively conducted by an RT.^5^–^7^,^9^

Internationally, strong evidence exists to support RT participation in review clinics.^1^,^6^–^10^ While information is available regarding the scope of practice and capabilities of RTs in performing reviews in the United Kingdom, there is still much variation in implementation between centres,^1^,^14^ largely due to differences in local need. There is a lack of data in the Australian RT environment to support the practice in the Australian context, although it may become more common in Australia in the future with demographic and workforce changes^15^,^16^ and with the increasing number of new cancer cases.^17^ Therefore, the purpose of this project was to investigate the feasibility of RTs undertaking a greater role in treatment review clinics at Calvary Mater Newcastle, New South Wales. Feasibility was determined according to the number of MIs required in treatment reviews and the attitudes and opinions of staff members about RTs performing treatment reviews.

## Methods

The Radiation Oncology Department at Calvary Mater Newcastle is equipped with five linear accelerators and has 80 clinical staff comprising consultant ROs, RO registrars, RTs, and nurses. The department treats an average of approximately 1900 patients annually.

An audit of 200 treatment reviews was undertaken in which the frequency of MI required of the RO was recorded using an observation checklist, the design and content of which was informed by previous studies and commentaries.^9^,^11^ The attitudes and opinions of clinical staff towards RT capability to participate in review clinics was assessed using the staff survey based on that used by Shi et al.^9^ Both measures were assumed to be of equal importance to determine feasibility.

For greater involvement of RTs in review clinics to be considered feasible required less than 35% MI across all observations made in the audit of the review clinics. The 35% level was used because it was the level of overall MI validated in the study performed by Shi et al.,^9^ while Grady and Back^11^ found an overall MI rate of 34% in a replication study. Both of those studies were performed at large metropolitan teaching hospitals similar to the Calvary Mater Newcastle. For the staff survey no particular target benchmark was established but it was considered that levels of agreement about RT capabilities approaching 100% should be achieved for feasibility to be recommended. Shi et al.^9^ found that RTs themselves were significantly more positive about RT participation in treatment review clinics than ROs. The ROs' perception of the RTs' capability was, therefore, considered to be of particular interest and an important element in addressing the question of feasibility.

### Audit of review clinics

The audit of review clinics was conducted by two observers, both RTs, who, combined, had 28 years clinical experience between them. They observed 200 separate reviews over a 6-month period between June and November 2011, a similar number to that observed in comparable audits.^9^,^11^ Treatment reviews were chosen from a convenience sample of patients receiving radiation therapy at the time, with a view to include a range of cancer sites, both palliative and radical treatments, and to accommodate the rostering of the observers. Over the 6-month period of the audit there was a total of 2941 scheduled review clinic appointments in the department for 746 patients. Hence, the 200 observed reviews were less than 7% of the total number of clinics during those 6 months. The sample of 200 reviews observed was broadly representative of the department's case mix.

The observation checklist was used to record the frequency of MI and non-MI-related procedures undertaken during the clinic. MI undertaken by ROs included writing a drug prescription, medical certificate or referral or ordering a treatment break. Non-MI included tasks such as performing a treatment toxicity assessment, providing information on treatment side effects, answering treatment technique questions, and providing emotional support.

Only one observer was present at each review clinic so, to help ensure inter-observer reliability, the first 24 treatment reviews were jointly coded and discussed to arrive at consensus, after which amendments were made to the observation checklist as required. Observation data were analysed with Statistical Package for Social Sciences (SPSS) version 19 for Windows (IBM, New York, NY) to obtain standard descriptive statistics.

### Staff survey

Questionnaires were distributed to 80 clinical staff members in August 2011, to be returned within 4 weeks. The 16 closed-ended questions included whether RTs were willing to participate in treatment reviews and whether ROs would be willing to delegate this role (see Table 4). There were 14 statements about how capable RTs would be “after suitable training and with the aid of established protocols” to perform specific treatment review tasks. The statements were based on those used by Shi et al.^9^ The closed-ended questions could be answered (either agree or disagree) and data were analysed using SPSS version 19 for Windows using standard descriptive statistics.

Two open-ended questions gave respondents an opportunity to freely express opinions regarding RT participation in review clinics and invited suggestions for implementation. They were analysed thematically after being transcribed verbatim from handwritten responses.

The project was exempted from ethics approval as a quality improvement exercise by the Hunter New England Human Research Ethics Committee. Permission was obtained from the ROs and patients before the observation of review clinics and no changes in practice or new procedures were introduced. All observation checklists and questionnaires were de-identified for data entry.

## Results

### Audit of review clinics

Eighty-two per cent of the patients were undergoing radical treatment and the remainder of them were for palliative needs. Male patients made up 63% of the sample and 70% were over 60 years of age. Overall, MI was required in 59% (*n* = 118) of the audited treatment reviews, with different MI rates for different treatment sites, the lowest being for breast (33%) and prostate (28%) cancers (Table 1). The highest rates of MI were for head and neck (93%) and gynaecological (91%) cancers.

**Table 1:** Breakdown of treatment review clinics requiring medical intervention (MI) and no MI according to the site of the cancer being treated

Treatment site	MI required *n* (%)	No MI required *n* (%)	Total clinics *n* (%)
Head and neck	41 (93)	3 (7)	44 (22)
Prostate	11 (28)	29 (73)	40 (20)
Chest	18 (78)	5 (22)	23 (12)
Rectum	13 (59)	9 (41)	22 (11)
Breast	7 (33)	14 (67)	21 (11)
Brain	8 (73)	3 (27)	11 (6)
Gynaecological	10 (91)	1 (9)	11 (6)
Bladder	3 (33)	6 (67)	9 (5)
Superficial	2 (33)	4 (67)	6 (3)
Bone metastases	2 (40)	3 (60)	5 (3)
Pelvis	2 (50)	2 (50)	4 (2)
Abdomen	1 (50)	1 (50)	2 (1)
Extremity	0 (0)	2 (100)	2 (1)
Total clinics	118 (59)	82 (41)	200 (100)

**Figure 1:**
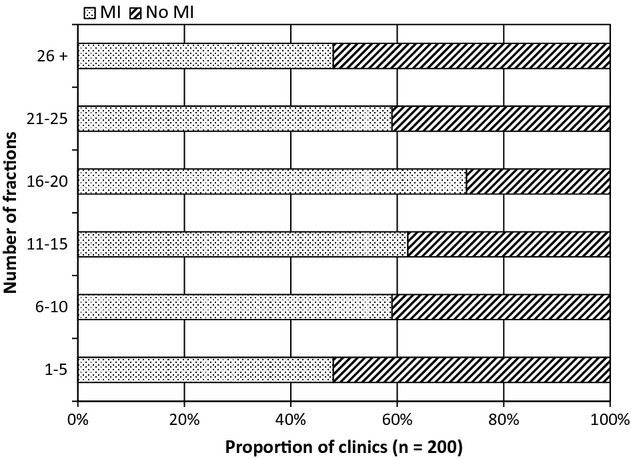
Bar chart showing the relationship between the numbers of fractions received and the rate of medical intervention (MI) required during treatment review clinics.

The required MI rate also varied over the course of patients' treatment according to how many treatment fractions they had received, as shown in Figure 1. The rate of MI was about 50% after the first five fractions and increased to its highest level of 73% for patients in their 4th week of treatment (fractions 16–20). Rates then declined again to approximately 50% for patients who had received 26 or more fractions.

The types of MI and non-MI required, their frequency, and the rate at which they were observed are shown in Tables 2 and 3. There were more than three times as many non-MIs as MIs observed and for those treatment reviews in which MI was noted there was an average of 1.4 MIs carried out. Writing a prescription and performing a physical examination were the most common forms of MI required, both occurring in 26% of reviews. The most common form of non-MI was toxicity assessment, performed in 94% of the observed treatment reviews, with discussion and education of treatment side effects the next most common, at 69%. The other non-MIs were much less common, occurring in 25% or less of the reviews.

**Table 2:** Frequency of medical interventions observed

Intervention	Frequency	% of clinics
Prescription given	52	26
Physical exam performed	52	26
Diagnostic or pathology tests ordered	26	13
Complex medications discussed	17	9
Medical certificate given	12	6
Referral given	4	2
Prescription altered	2	1
Treatment break ordered	1	1
Physiotherapy referral given	0	0
Total medical interventions	166	

**Table 3:** Frequency of non-medical interventions observed

Intervention	Frequency	% of clinics
Toxicity assessment performed	188	94
Side effects education and advice given	137	69
Chemotherapy/hormones discussed	49	25
Information on patient's cancer discussed	41	21
Unrelated medical issues discussed	40	20
Nutritional advice given	31	16
Treatment technique explained	24	12
Psychosocial support given	19	10
Referral to allied health given	5	3
Complementary and alternative medicine discussed	3	2
Other general cancer information discussed	2	1
Dressing ordered to be done by nurses	2	1
Total non-medical interventions	541	

### Staff survey

The overall survey response rate was 75% (*n* = 60). All but one of the respondents (a registrar) agreed RTs would be willing to participate in treatment review clinics (Table 4); however, all five consultant ROs indicated that they were not willing to delegate clinics to RTs. In contrast, all four of the RO registrars responded they would be willing to delegate review clinics to RTs.

**Table 4:** Capability statements about radiation therapists by decreasing strength of agreement by survey respondents (*n* = 60)

Radiation therapist capability statements	RTs (*n* = 44)	Nurses (*n* = 7)	ROs (*n* = 5)	Reg's (*n* = 4)
RTs, after suitable training and with the aid of established protocols, are capable of the following:				
• Using the common terminology/toxicity criteria scoring system to grade toxicities	44 (100%)	7 (100%)	5 (100%)	4 (100%)
• Answering treatment-related logistics questions (appointments etc.)	44 (100%)	7 (100%)	5 (100%)	4 (100%)
• Providing assurance to patients undergoing radiation therapy	44 (100%)	7 (100%)	4 (80%)	4 (100%)
• Answering treatment technique-related questions	44 (100%)	7 (100%)	5 (100%)	3 (75%)
• Answering radiotherapy side effects questions	44 (100%)	7 (100%)	2 (40%)	4 (100%)
• Providing information on the side effects a patient may experience	44 (100%)	7 (100%)	3 (60%)	3 (75%)
• Participating in treatment reviews	44 (100%)	3 (43%)	4 (80%)	3 (75%)
• Giving adequate nutrition advice	40 (91%)	1 (14%)	1 (20%)	4 (100%)
• Recommending drugs to treat standard side effects	36 (82%)	2 (29%)	1 (20%)	1 (25%)
• Answering general cancer-related questions	38 (86%)	3 (43%)	1 (20%)	0
• Providing information on the cancer a patient has	20 (45%)	3 (43%)	2 (40%)	0
• Answering complementary and alternative medicine-related questions	16 (36%)	1 (14%)	0	1 (25%)
• Deciding whether a patient should have a break from treatment	10 (23%)	2 (29%)	0	1 (25%)
• Answering general medicine-related questions	16 (36%)	1 (14%)	0	0
RTs are willing to participate in treatment reviews	44 (100%)	7 (100%)	5 (100%)	3 (75%)
ROs are willing to delegate treatment reviews	14 (32%)	5 (71%)	0	4 (100%)

Proportions of respondents in each category who responded affirmatively are rounded to the nearest whole percentage. RTs, radiation therapists; Nurses, both registered and enrolled nurses; ROs, consultant radiation oncologists; Reg's, radiation oncology registrars.

For the 14 statements about the RTs' capabilities in relation to treatment review clinics the highest level of agreement with the statements was found among the RTs, followed by the nurses, registrars, and then the consultant ROs. In Table 4, the 14 capability statements are ordered according to proportion of respondents in each occupational group who agreed with each statement. All respondents in all four groups agreed with the top two statements. All the RTs agreed with the top seven statements and all the nurses with the top six. At least some RTs and nurses agreed with all 14 statements, while very few of the consultants or registrars agreed with any of the lowest ranked statements. For 10 of the 14 statements, the registrars responded the same or more positively than consultants with regard to the RTs' capabilities.

Analysis of the two open-ended questions showed consultant ROs' concerns about potential double handling of complex cases, as well as of medico-legal implications. Registrars suggested RTs could review patients receiving treatment for breast or prostate cancer and would be good at toxicity assessment. While the main concern expressed by RTs was education and training, they also felt that RTs' closer contact with patients enables follow-up care to be provided. They also felt patients waited too long to be reviewed. Responses from the nurses indicated general disagreement with RTs participating in review clinics. They were unified in their view that the current format of review clinics works well and change is not warranted.

## Discussion

Neither of the two feasibility measures for greater involvement of RTs in treatment review clinics have been met in this study. Overall, the proportion of reviews in which MI was observed was well above the 35% mark set for feasibility to be considered. The overall MI rate was also higher in this study than that previously reported.^9^,^11^ This could be due to differences in the observation checklist and how it was used in this study compared to the other studies, although these differences were minor. The implications of the disparity are unclear and further investigation is recommended.

In this study, MI rates for breast and prostate cancer are lower than those for other cancer types and below the 35% level. The lower rate of MI for breast and prostate cancer supports previous work of Shi et al.^9^ and Grady and Back^11^ and suggests RT participation in clinics would be best suited to these treatment sites. With respective breast and prostate cancer MI rates of 33% and 28%, some patients would still require referral and consultation with their RO, although these rates could be reduced with education and experience of specialist RTs. High concordance in breast cancer patient treatment reviews between specialist RTs and ROs has been found elsewhere,^7^ further supporting the argument that RTs could be specifically educated and trained to participate in review clinics for this subset of patient presentations.

Because both breast and prostate cancers are relatively common cancer types, the reduction in RO routine workload could be substantial, permitting them more time to spend with patients with more complex disease and requiring higher levels of MI. Any implementation of RT participation in review clinics would need to be from an integrated team-based approach,^9^ with ROs having more time available for patients requiring MI, while RT and nurse practitioner teams could attend to patients whose treatment was proceeding according to expectations, with minimal complications.

The MI rate was highest for patients in their 4th week of treatment, occurring in almost 75% of treatment reviews. Prior to the 16th fraction and after the 20th fraction rates of MI were lower. In the period of fractions from 1 to 5, when treatment side effects are early and less frequent, about 50% reviews required MI. After the 25th fraction, when late side effects and comorbidities are being appropriately managed, MI rates again fell to about 50%. Although at no stage did the MI rate fall to the 35% level where feasibility might be considered, if the future treatment review clinical practice model was to be changed to include RT participation, then perhaps the best clinics to target would be early and late in the treatment period when MI is least likely to be needed.

The survey responses obtained from the RTs were similar to those reported by Colyer,^5^ with RTs seeing themselves as more accessible and closer to patients, facilitating better communication, and enabling participation in review clinics. However, the low level of support expressed by the consultant ROs and nurses was not entirely unexpected as it is similar to that reported by White et al.^18^ in Hong Kong, where implementation of RT-led clinics was hindered by a lack of willingness and support from other professionals. ROs were unwilling to delegate duties and nurses were resistant to the idea of sharing competencies with RTs.^18^ In Hong Kong, this was attributed to greater medical dominance than that reported in previous UK studies,^5^,^18^,^19^ although the reasons are likely to be much more complex and diverse and reflect ROs' concerns for their patients' well-being and a lack of confidence in RTs to perform treatment reviews. While in the study by Shi et al.^9^ RTs and ROs were both supportive of RT participation in review clinics, RTs felt significantly more positive about their capability; in the study by Grady and Back^11^ ROs were more positive about RTs' capabilities than the RTs themselves. The reason for these discrepancies is not known but may be related to local conditions and personalities.

It is unclear if the survey responses are representative of the wider Australian workforce. The results differ from those obtained in another Australian institution where a similar survey was conducted and RO support for RT participation was higher.^11^ This finding may have been influenced by differences in the questionnaire, with this study having qualified all capability statements in the context of the RTs having undertaken “suitable training” and being aided by “established protocols,” whereas the previous study did not consider these criteria. The differences suggest a need for further investigation of the factors that influence the attitudes of ROs and RTs in relation to this and other “advanced practice” roles, as well as the educational needs and expectations for RTs to undertake such roles.

Establishment of extended scope of practice within non-medical professions is difficult and fraught with legal and ethical challenges.^19^,^20^ These challenges may influence the ROs' reluctance to delegate tasks that they traditionally perform, as reflected by ROs' concerns about medico-legal implications expressed in the survey. There is need for further qualitative research into ROs' concerns. Acharya et al.^19^ suggest that this may be dealt with by providing support and training and targeting RTs who have substantial clinical experience to underpin their extended role. Furthermore, it can be argued that before instigating any RT participation in review clinics, collaboration with ROs in defining the guidelines and protocols for practice would be essential. Clear role delineation would minimize potential for duplication, which was another concern reflected in the surveys, and help to minimize friction and inefficiency within departments.^9^

A legal barrier also exists to changing the practice model because of current Medicare legislation, which stipulates that to attract a Medicare benefit radiation oncology services must be provided by a medical specialist.^21^ There is no provision at this time for such services to be delegated to non-medical practitioners; thus, for change to take place, consideration must be given to legislative changes. This complex discussion is beyond the scope of this article.

One hundred per cent of the survey respondents indicated that they thought RTs are capable of using the common toxicity criteria (CTC) scoring system to grade treatment toxicities, which was the most common non-MI observed. Additionally, RTs are well equipped with their current education and experience to explain treatment techniques, again with 100% agreement by RTs, ROs, and nurses that RTs are capable in this area. In other capabilities, however, such as answering questions about side effects or giving advice about nutrition or complementary and alternative medicines, RTs would need to undergo considerable further education and training for their participation in review clinics to be feasible. Interventions such as giving general medical advice or advising if a patient needs a break from treatment should remain the role of the RO.

Implementation of a shared care approach may be more acceptable in cancer centres located in regional or rural areas, where recruitment and retention of ROs is difficult. Similar extended scope roles in nursing duties have developed in rural areas.^22^ RTs may be able to complement the role of ROs who are sole practitioners or in part-time positions in regional radiotherapy centres.^23^ The shared care model could vary depending on local needs, perhaps with RTs and nurses working together to broaden the local knowledge and skill base.^10^ Where ROs are in short supply, consultation with the RO could take place using information technology and fast broadband connections. Such practice changes are likely, therefore, to be driven by local need and resource availability.

Although this small study achieved the aim of investigating the feasibility of implementing RT participation in treatment review clinics at one particular centre, there are some limitations that must be acknowledged. The treatment reviews observed were a small convenience sample and it may be improbable that the results will be generalizable for all clinics at all cancer centres. It is acknowledged that the 35% feasibility benchmark is dependent on the items included on the MI checklist and is perhaps not an ideal measure. Furthermore, there was no correlation of MI rate and disease stage at diagnosis, which could reasonably be expected to have a substantial effect. It could also be argued that the definition of MI is somewhat limiting and may be subjective. Such factors should be taken into account in the design of any future, similar studies.

In the survey, the closed questions were all positively phrased, which introduces potential for acquiescence bias. Also, for simplicity, a dichotomous scale was used (agree or disagree), with the disadvantage of preventing participants from responding less adamantly. The survey sample size for ROs, registrars, and nurses was small and a larger scale survey of radiation oncology staff would provide a clearer understanding of the opinions from the broader Australian oncology department workforce. It would also be appropriate in future research to examine the possible economic impact of changes in the practice model, the medico-legal issues, and the patients' perceptions of such models of service provision.

## Conclusions

The results of this study do not support the immediate introduction of RT participation in radiation therapy treatment review clinics in this department, because of high levels of MI required in most clinics and equivocal support from ROs and other clinical staff. There are, however, some results that suggest possible changes to future practice models and it is hoped these findings will stimulate discussion regarding RTs participating in treatment review clinics.

Accepting the fact that ROs' workload has increased at a faster rate than the increase in workforce, and that this pattern is unlikely to change in the foreseeable future, there will be a need for the development of new practice models that involve greater sharing of the workload across professional boundaries, with interprofessional collaboration and teamwork as important goals. On the basis of some of the findings of this study and of other studies in this field, it seems that future participation of RTs in review clinics, as well as other extended scope of practice roles, could reasonably be considered in restructuring the health workforce in the delivery of radiation oncology services. However, it is also clear that such changes cannot take place without considerable planning and re-education of the workforce to overcome current barriers to new models of care and to meet future needs. It can be argued that planning should be taking place now, rather than waiting and allowing the workloads to increase to even more unsustainable levels, with the consequent risk to the quality of patient services.
